# Supplementary material for: Light‐Activated Nucleic Acid Amplification Systems Using Photo‐Caged DNA Polymerase or Primers
Source: Adv Genet (Hoboken). 2026 May 20;7(2):e00055. doi: 10.1002/ggn2.202500055 (PMC13374820; doi:10.1002/ggn2.202500055)
Supplement: Supplementary file 1 — Supporting File: ggn270037‐sup‐0001‐SuppMat.docx. [file GGN2-7-e00055-s001.docx]

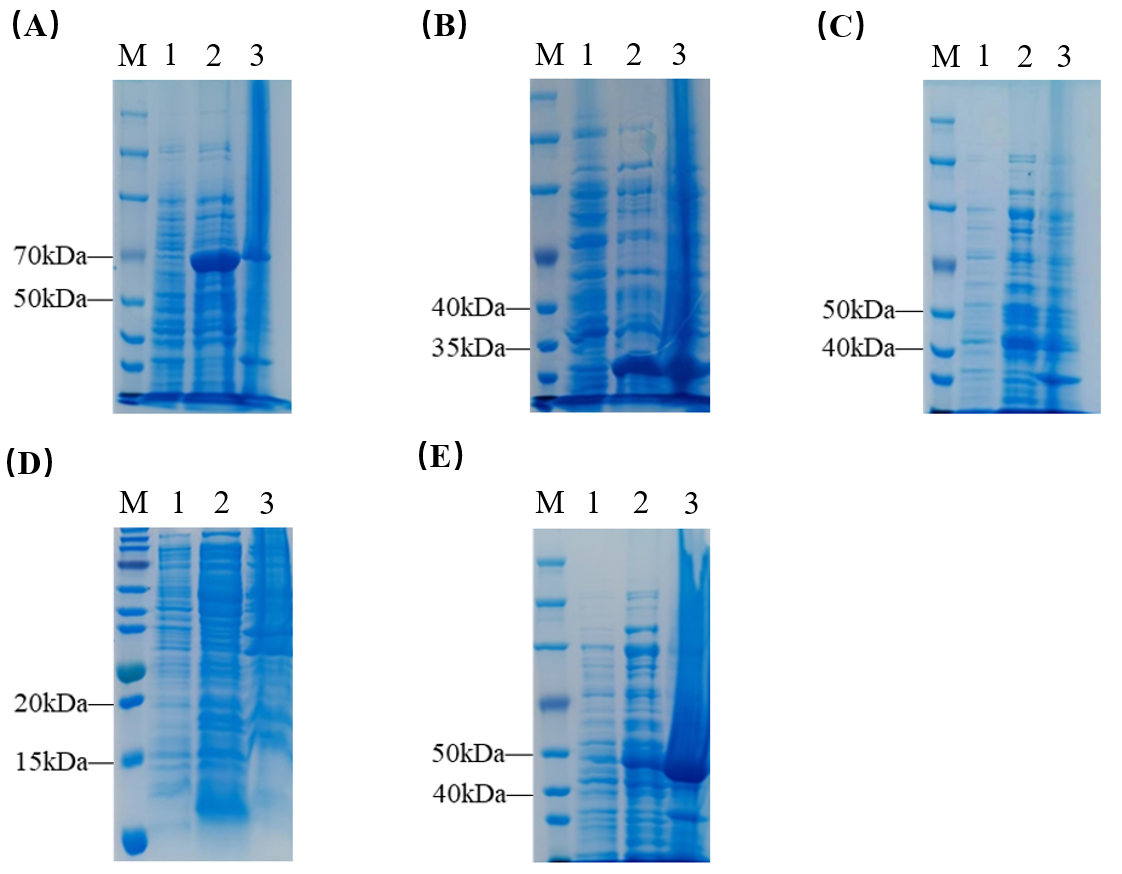


**Figure S1**. SDS-PAGE analysis of protein expression for five key RAA components in *E. coli*.
**(A)** DNA polymerase; **(B)** single-stranded DNA-binding protein (gp32); **(C)** recombinase (UvsX); **(D)** co-recombinase (UvsY); **(E)** exonuclease III (Exo III). For each protein, **Lane 1**: total lysate before IPTG induction; **Lane 2**: soluble fraction after IPTG induction; **Lane 3**: insoluble fraction (inclusion bodies) after IPTG induction.


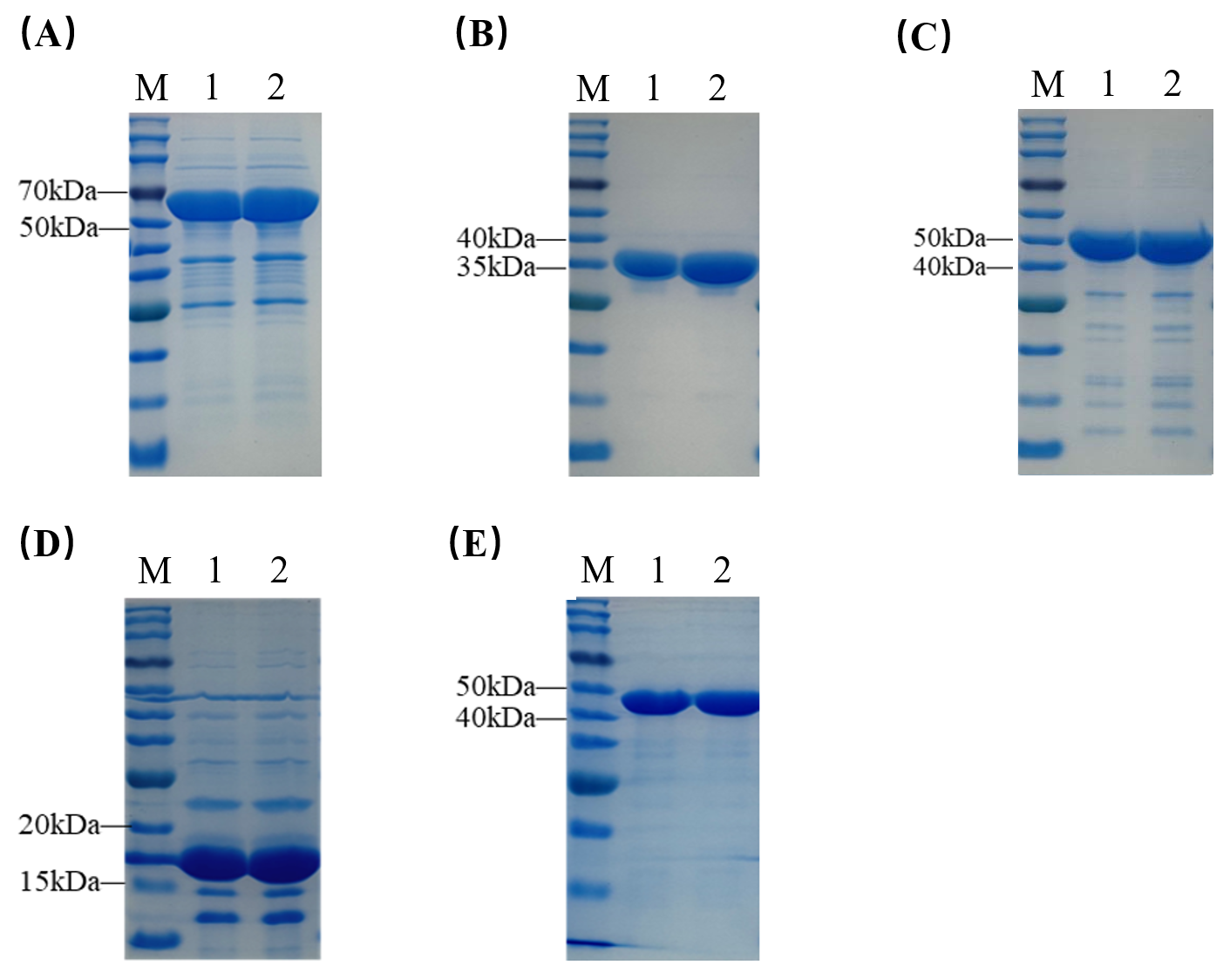


**Figure S2**. SDS-PAGE analysis of purified recombinant proteins used in RAA system.
**(A)** DNA polymerase; **(B)** single-stranded DNA-binding protein (gp32); **(C)** recombinase (UvsX); **(D)** co-recombinase (UvsY); **(E)** exonuclease III (Exo III). For each protein, **Lane 1**: purified protein sample before dialysis; **Lane 2**: protein sample after dialysis.


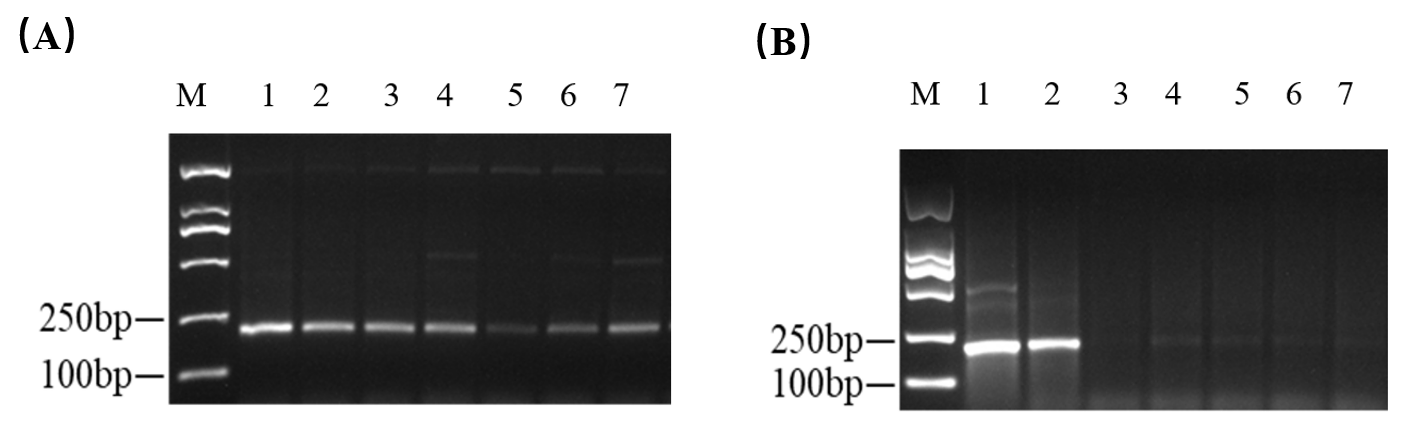


**Figure S3**. Optimization of the Conjugation Ratio Between Azide-Containing DNA Polymerase and DBCO-Modified Single-Stranded DNA (ssDNA) Blocker.
Assessment of the optimal molar ratio for the strain-promoted azide–alkyne cycloaddition (SPAAC) reaction between azide-functionalized DNA polymerase and DBCO-modified ssDNA blocker used to generate photocaged polymerase.
**(A)** Conjugation performed at a 1:2 molar ratio (enzyme:ssDNA); **(B)** Conjugation performed at a 1:3 molar ratio. After conjugation, the activity of the resulting photocaged polymerase was evaluated in the RAA system with and without UV light activation.
In both panels, **Lane 1–2**: amplification products using wild-type (uncaged) DNA polymerase as positive controls; **Lane 3**: photocaged polymerase without UV irradiation, showing suppressed amplification; **Lanes 4–5**: photocaged polymerase after 180 seconds of UV activation, showing partial restoration of amplification; **Lanes 6–7**: photocaged polymerase after 300 seconds of UV activation, demonstrating enhanced recovery of enzymatic activity.
